# Neurotropin^®^ Accelerates the Differentiation of Schwann Cells and Remyelination in a Rat Lysophosphatidylcholine-Induced Demyelination Model

**DOI:** 10.3390/ijms19020516

**Published:** 2018-02-08

**Authors:** Hozo Matsuoka, Hiroyuki Tanaka, Junichi Sayanagi, Toru Iwahashi, Koji Suzuki, Shunsuke Nishimoto, Kiyoshi Okada, Tsuyoshi Murase, Hideki Yoshikawa

**Affiliations:** 1 Department of Orthopaedic Surgery, Osaka University Graduate School of Medicine, 2-2 Yamadaoka, Suita, Osaka 565-0871, Japan; go_go_475_going_my_way@yahoo.co.jp (H.M.); junby1023@gmail.com (J.S.); kurobuchi0918@gmail.com (T.I.); kokada@hp-mctr.med.osaka-u.ac.jp (K.O.); tmurase-osk@umin.ac.jp (T.M.); yhideki@ort.med.osaka-u.ac.jp (H.Y.); 2 Department of Orthopaedic Surgery, Kansai Rosai Hospital, 3-1-69 Inabaso, Amagasaki, Hyogo 660-0064, Japan; kouji_szk@hotmail.co.jp (K.S.); tennishun37@gmail.com (S.N.); 3 Medical Center for Translational and Clinical Research, Osaka University Hospital, 2-15 Yamadaoka, Suita, Osaka 565-0871, Japan

**Keywords:** Neurotropin^®^, Schwann cells, differentiation, AKT, extracellular signal-regulated kinase1/2, remyelination, peripheral nerve, regeneration

## Abstract

Neurotropin^®^ (NTP), a non-protein extract of inflamed rabbit skin inoculated with vaccinia virus, is clinically used for the treatment of neuropathic pain in Japan and China, although its effect on peripheral nerve regeneration remains to be elucidated. The purpose of this study was to investigate the effects of NTP on Schwann cells (SCs) in vitro and in vivo, which play an important role in peripheral nerve regeneration. In SCs, NTP upregulated protein kinase B (AKT) activity and Krox20 and downregulated extracellular signal-regulated kinase1/2 activity under both growth and differentiation conditions, enhanced the expression of myelin basic protein and protein zero under the differentiation condition. In a co-culture of dorsal root ganglion neurons and SCs, NTP accelerated myelination of SCs. To further investigate the influence of NTP on SCs in vivo, lysophosphatidylcholine was injected into the rat sciatic nerve, leading to the focal demyelination. After demyelination, NTP was administered systemically with an osmotic pump for one week. NTP improved the ratio of myelinated axons and motor, sensory, and electrophysiological function. These findings reveal novel effects of NTP on SCs differentiation in vitro and in vivo, and indicate NTP as a promising treatment option for peripheral nerve injuries and demyelinating diseases.

## 1. Introduction

Peripheral nerve injury (PNI) has a high incidence rate of 2.8% of all traumas [1]. With current therapies, nerve regeneration after injury is still a challenge. In general, the peripheral nervous system (PNS) has a far greater capacity to regenerate towards its original target and recover functionally in response to injuries unlike the central nervous system (CNS). However, regeneration after PNI is not necessarily complete, even when the injured nerves are microsurgically repaired. This often results in delayed recovery of motor and/or sensory function in patients who have sustained nerve injuries. This is particularly the case in older patients, and after chronic denervation or injuries to large peripheral nerve trunks, such as the brachial plexus and the lumbar plexus [2,3,4,5]. Successful regeneration of injured peripheral nerves is challenging but has been an area of intense research.

In the PNS, Schwann cells (SCs) are responsible for myelin formation, which contributes to axonal protection and allows saltatory conduction [6,7]. Nerve regeneration is a complex and continuous process. Upon PNI, myelin and axons distal to the injury site display Wallerian degeneration which mainly contains degeneration of distal axons and subsequent de-differentiation, proliferation, differentiation, and myelin formation of the ensheathing SCs [8]. These findings suggest that SCs are very important for nerve regeneration after PNI.

Neurotropin^®^ (NTP) is a non-protein extract derived from the inflamed skin of rabbits inoculated with vaccinia virus. The safety of NTP has already been established, having been widely used in Japan and China for more than 50 years for the treatment of neuropathic pain, such as post-herpetic neuralgia, hyperesthesia of subacute myelo-optic neuropathy, and chronic pain such as lower back pain and cervico-omo-brachial syndrome [9,10,11,12,13]. In the CNS, the analgesic effect of NTP is considered to be mediated by activation of descending-pain inhibitory pathways via the serotonergic and noradrenergic systems projecting from supraspinal sites to the spinal dorsal horn [11]. In addition, NTP inhibits the release of bradykinin, which plays an important role in evoking pain sensation in the PNS [14]. However, there have been no reports on the effect of NTP on peripheral nerve regeneration.

We previously reported that NTP inhibits extracellular signal-regulated kinase (ERK) activity and demyelination induced by chronic constriction injury of a mouse sciatic nerve [15]. Some studies have identified that activation of protein kinase B (AKT) and mitogen activated protein kinase (MAPK) including ERK pathways play a role in SCs proliferation, differentiation, and dedifferentiation [16,17,18,19]. In particular, the sustained activity of AKT signaling is essential for SCs myelination [16], while ERK signaling negatively regulates myelination [18]. These findings remind us of the possibility that NTP promotes nerve regeneration by accelerating the differentiation of SCs after PNI. In this study, we assessed the influences of NTP on SCs differentiation in vitro and remyelination in a rat sciatic nerve focal demyelination model in vivo.

## 2. Results

### 2.1. NTP Increases AKT Activity and Reduces ERK1/2 Activity in SCs under Growth Conditions

**Figure 1:**
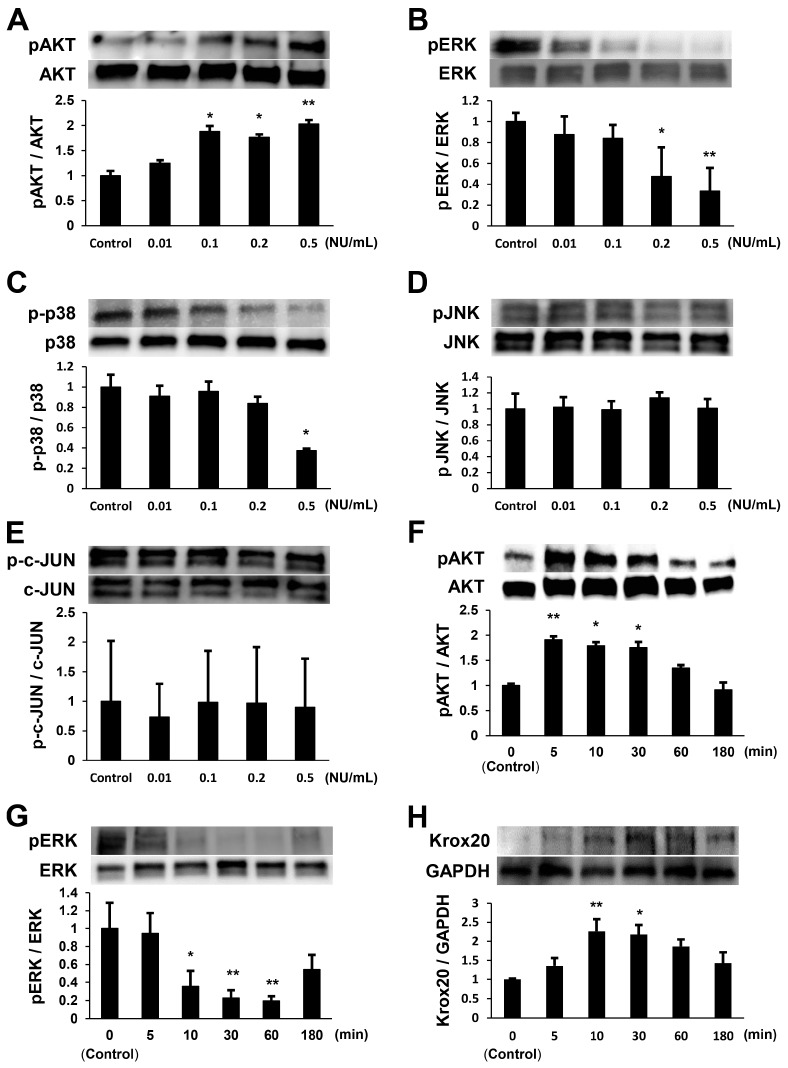

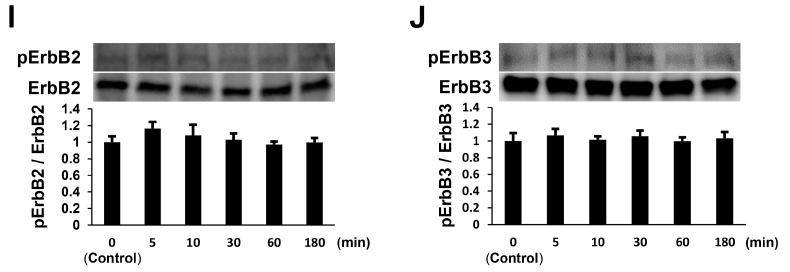
Neurotropin (NTP) increases protein kinase B (AKT) activity and reduces ERK1/2 activity in Schwann cells (SCs) under growth conditions. (**A**–**E**) SCs were cultured with NTP at concentrations of 0.01–0.5 NU/mL for 1 h in growth medium. Representative western blotting images (top) and quantified expression levels (bottom) of phosphorylated/total AKT, ERK1/2, p38, JNK, and c-JUN using densitometric analysis are shown. NTP increased AKT (**A**) activity and reduced ERK1/2 (**B**) and p38 (**C**) activities in SCs in a dose-dependent manner, but did not affect JNK (**D**) and c-JUN (**E**) activities. (**F**–**J**) SCs were cultured for up to 180 min in growth medium with NTP at a concentration of 0.5 NU/mL. Representative western blotting images (top) and quantified expression levels (bottom) of phosphorylated AKT, phosphorylated ERK, and Krox20 expression in SCs using densitometric analysis are shown. The quantified expression level was normalized to the total AKT and ERK expression level for phosphorylated AKT and ERK, or normalized to the GAPDH expression level for Krox20. GAPDH was used as an internal control. (**F**) The activity of AKT was significantly enhanced, compared with the control and reached a peak 5 min after NTP addition. (**G**) The activity of ERK1/2 was most reduced compared with the control at 60 min after the addition of NTP. (**H**) The expression of Krox20 was significantly enhanced, compared with the control and reached a peak 10 min after NTP addition. Representative western blotting images (top) and quantified expression levels (bottom) of phosphorylated/total ErbB2 and ErbB3 using densitometric analysis are shown. NTP did not affect ErbB2 (**I**) and ErbB3 (**J**) activities. Significance was determined by one-way ANOVA followed by Dunnett’s test. Graphs show mean ± SD and results are representative of more than three independent experiments; * *p* < 0.05, ** *p* < 0.01 compared with the control.

To determine whether NTP influences AKT and MAPK (ERK1/2, p38, and c-Jun-N-terminal kinase (JNK)) activities in SCs, we detected their activation in SCs cultured for 1 h with NTP at concentrations of 0.01 to 0.5 Neurotropin unit (NU)/mL in growth medium. We observed that NTP significantly increased AKT activity (Figure 1A) and suppressed ERK1/2 (Figure 1B) and p38 (Figure 1C) activities in SCs in a dose-dependent manner. JNK (Figure 1D) and c-JUN (Figure 1E) activation was not detected at any concentration. The results of these experiments suggest that the axis of p38/c-JUN is not a main downstream target of NTP. SCs were then cultured with NTP (0.5 NU/mL) for up to 180 min in growth medium. NTP temporarily led to a 1.91 ± 0.06-fold stronger activation of AKT 5 min after NTP addition than that observed in the control (Figure 1F), and a 0.19 ± 0.05-fold weaker activation of ERK1/2 at 60 min than that in the control (Figure 1G). The change in AKT and ERK1/2 activities described above led us to hypothesize that NTP would induce SCs to differentiate. Therefore, we next evaluated the expression of Krox20, one of the major promyelinating transcription factors [6,20]. NTP increased the expression of Krox20 maximally at 10 min 2.26 ± 0.32-fold over that in the control in growth medium (Figure 1H). Neuregulin-1 (NRG1) provides a key axonal signal that regulates Schwann cell proliferation, migration, and myelination through binding to ErbB2/3 receptors, which activate various signaling cascades including AKT and ERK pathways [21,22,23]. However, the activation of both ErbB2 and ErbB3 was not detected in growth medium after NTP treatment (Figure 1I,J). These results clearly demonstrate that NTP increases AKT activity and reduces ERK1/2 activity in SCs in growth medium, albeit temporarily.

### 2.2. NTP Increases AKT Activity and Reduces ERK1/2 Activity in SCs under Differentiation Conditions

**Figure 2:**
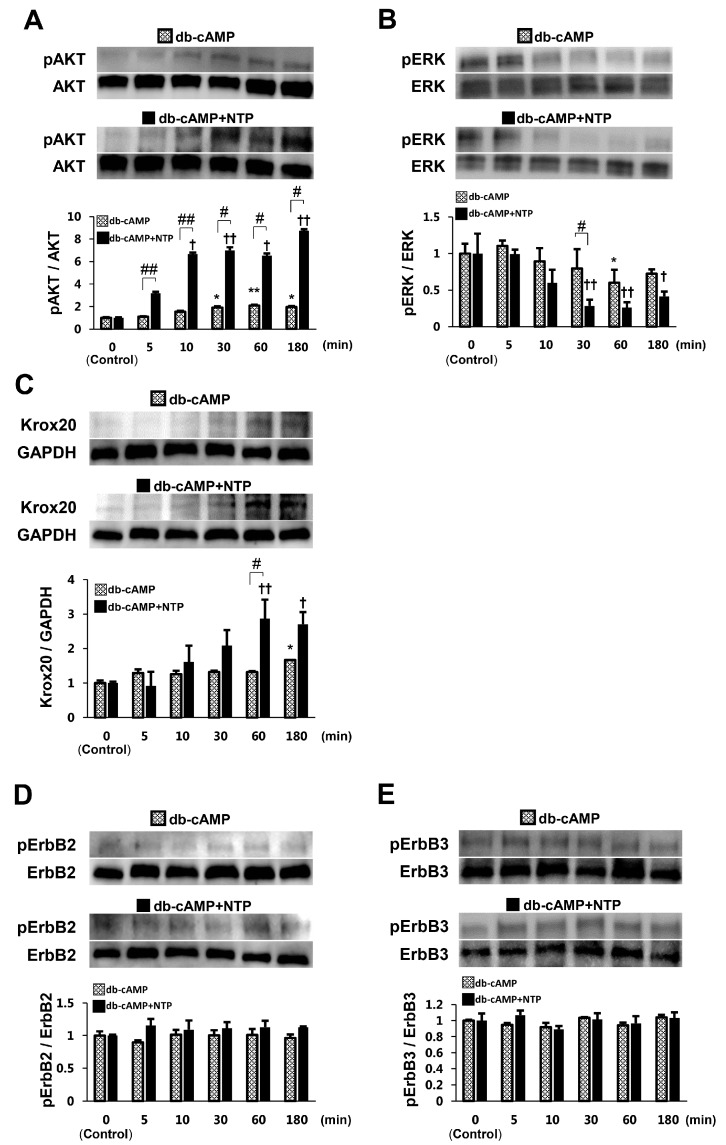
NTP increases AKT activity and reduces ERK1/2 activity in SCs under differentiation conditions. (**A**–**E**) SCs were cultured for 180 min in differentiation medium containing db-cAMP, with or without NTP at a concentration of 0.5 NU/mL. Representative western blotting images (top) and quantified expression levels (bottom) of phosphorylated AKT, phosphorylated ERK, and Krox20 expression in SCs using densitometric analysis are shown. The quantified expression level was normalized to the total AKT and ERK expression level for phosphorylated AKT and ERK, or normalized to the GAPDH expression level for Krox20. GAPDH was used as an internal control. (**A**) AKT activity was significantly enhanced compared with the control and reached a peak at 180 min after the addition of NTP. (**B**) ERK1/2 activity was significantly reduced compared with the control and was most reduced at 60 min. (**C**) The expression of Krox20 was significantly enhanced compared with the control and reached a peak at 60 min after NTP addition. Representative western blotting images (top) and quantified expression levels (bottom) of phosphorylated/total ErbB2 and ErbB3 using densitometric analysis are shown. NTP did not affect ErbB2 (**D**) and ErbB3 (**E**) activities. Significance was determined by one-way ANOVA followed by a post hoc Student’s *t*-test or Dunnett’s test. Graphs show mean ± SD and results are representative of more than three independent experiments; * *p* < 0.05, ** *p* < 0.01, † *p* < 0.05, †† *p* < 0.01 compared with the 0 minutes control and # *p* < 0.05, ## *p* < 0.01 compared with the no NTP control.

To estimate whether NTP affects AKT and ERK1/2 activities in SCs under differentiation conditions, we first examined their activation in SCs cultured in differentiation medium containing dibutyryl cyclic AMP (db-cAMP), a membrane-permeable derivative of cAMP. cAMP signaling is sufficient to potently induce myelin gene expression and allows SCs to transition into a myelinating stage [24]. We observed that administration of db-cAMP more strongly activated AKT (Figure 2A) and inactivated ERK1/2 (Figure 2B) at 60 min in comparison with the control. In addition, we investigated the expression of Krox20 and the activation of both ErbB2 and ErbB3 in differentiation medium. The expression of Krox20 was significantly enhanced at 180 min after db-cAMP addition, compared with the control (Figure 2C). The activation of both ErbB2 and ErbB3 was not detected in differentiation medium (Figure 2D,E). SCs were next treated with NTP (0.5 NU/mL) for up to 180 min in differentiation medium containing db-cAMP. We found that NTP temporarily led to an 8.74 ± 0.14-fold stronger activation of AKT at 180 min than that observed in the control (Figure 2A) and a 0.26 ± 0.07-fold weaker activation of ERK1/2 at 60 min than that in the control (Figure 2B). NTP increased the expression of Krox20 maximally at 60 min 2.87 ± 0.55-fold over that in the control in differentiation medium (Figure 2C). The activation of both ErbB2 and ErbB3 was not detected in differentiation medium after NTP treatment (Figure 2D,E). These results show that NTP increases AKT activity and suppresses ERK1/2 activity in SCs in differentiation medium.

### 2.3. NTP Does Not Affect SCs Proliferation

**Figure 3:**
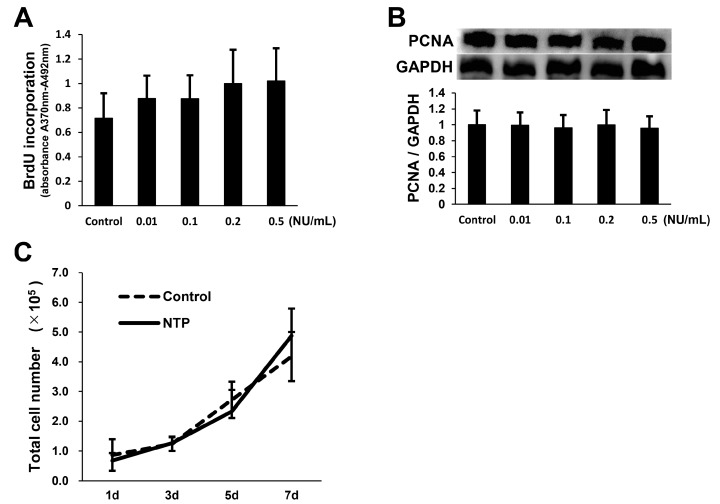
NTP does not affect SCs proliferation. (**A**) SCs were cultured with NTP at concentrations of 0.01–0.5 NU/mL in growth medium for 2 h after the addition of BrdU. The BrdU incorporation rate of SCs was detected by a BrdU uptake assay. (**B**) SCs were cultured with NTP at concentrations of 0.01–0.5 NU/mL for 1 h in growth medium. Representative western blotting images (top) and quantified expression levels (bottom) of PCNA using densitometric analysis are shown. GAPDH was used as an internal control. (**C**) SCs were maintained in growth medium for 7 days in the presence or absence of 0.5 NU/mL NTP and cell counting was performed at days 1, 3, 5, and 7. (**A**–**C**) There were no significant differences in the values between NTP and the control groups. The data were analyzed by one-way ANOVA followed by a post hoc Student’s *t*-test or Dunnett’s test. Graphs show mean ± SD and results are representative of four independent experiments.

We next evaluated the effect of NTP on SCs proliferation. SCs were treated with 5-bromo-2-deoxyuridine (BrdU) in the presence or absence of NTP (0.01–0.5 NU/mL) and there was no change of BrdU uptake at any concentration (Figure 3A). A well-defined cell-proliferation marker, proliferating cell nuclear antigen (PCNA), was further analyzed by western blotting. No statistically significant differences were observed in PCNA protein levels (Figure 3B). SCs were cultured with or without NTP (0.5 NU/mL) for 7 days, and cell number counting was performed thereafter. The results indicated that no significant differences were found between the control and NTP groups on days 1, 3, 5, and 7 (Figure 3C). These findings suggest that NTP does not stimulate SCs proliferation.

### 2.4. NTP Accelerates the Differentiation of SCs In Vitro

**Figure 4:**
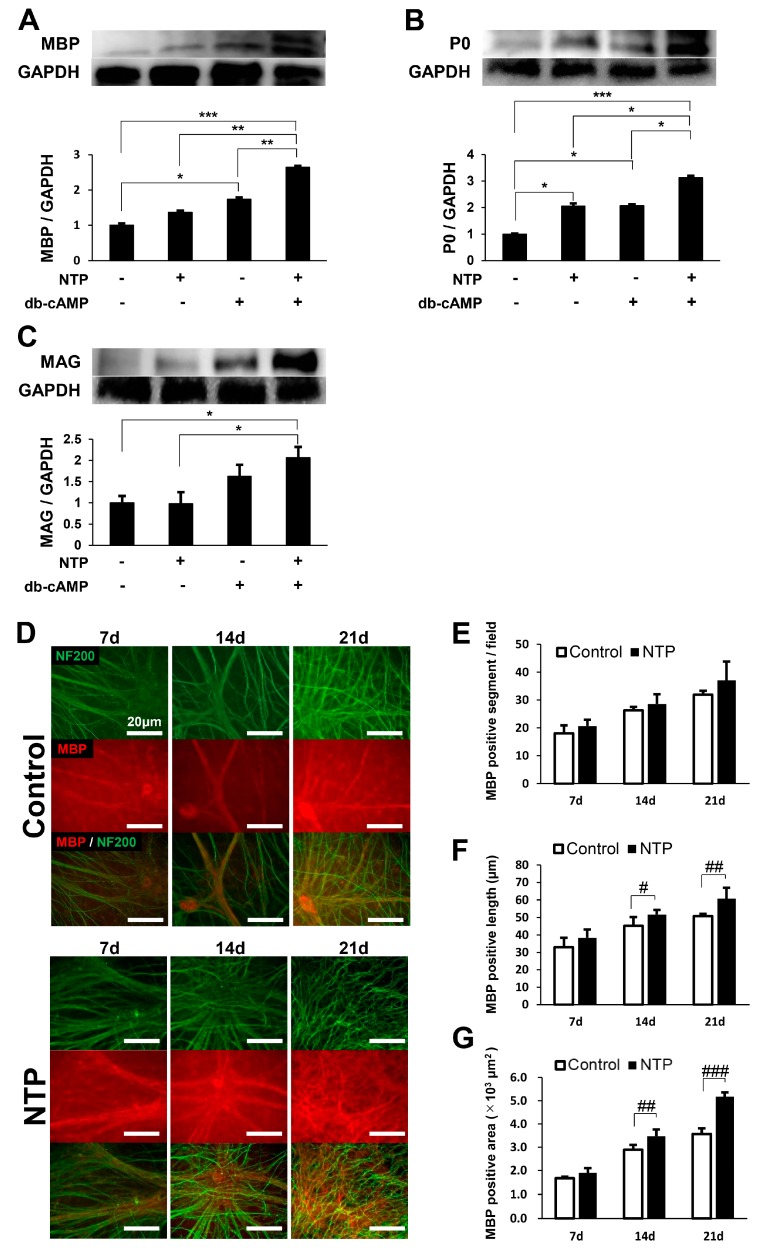
NTP accelerates the differentiation of SCs in vitro. (**A**–**C**) SCs were maintained in growth medium or differentiation medium containing db-cAMP for 72 h with or without NTP (0.5 NU/mL). Representative western blotting images (top) and quantified expression levels (bottom) of MBP, P0, and MAG using densitometric analysis are shown. GAPDH was used as an internal control. Significance was determined by one-way ANOVA followed by the Tukey–Kramer HSD test. Graphs show mean ± SD and results are representative of more than three independent experiments; * *p* < 0.05, ** *p* < 0.01, *** *p* < 0.001. (**D**–**G**) DRG/SC co-cultures were maintained with or without NTP (0.5 NU/mL) under differentiation conditions. (**D**) Representative fluorescence micrographs of co-cultures labeled with anti-MBP antibody (red) and anti-NF200 antibody (green) are shown at 7, 14, and 21 days after the induction of differentiation. Scale bar = 20 μm. The extent of myelination was quantified by measuring the number (**E**), length (**F**), and area (**G**) of MBP-positive segments. (**E**–**G**) Significance was determined by one-way ANOVA followed by a post hoc Student’s *t*-test. Graphs show mean ± SD and results are representative of five independent experiments; # *p* < 0.05, ## *p* < 0.01, ### *p* < 0.001 compared with the control.

We next focused on the effect of NTP on SCs differentiation and evaluated the expression of myelin-related proteins such as myelin basic protein (MBP), protein zero (P0), and myelin-associated glycoprotein (MAG) in both the growth and the differentiation media, with or without NTP (0.5 NU/mL). NTP significantly accelerated the expression of MBP in the differentiation condition but not in the growth condition (Figure 4A). In addition, the expression of P0 was significantly accelerated by NTP in both the growth and differentiation media (Figure 4B). NTP did not significantly promote the expression of MAG in any medium, although a trend to increased expression was observed in the differentiation medium (Figure 4C).

To further explore the effect of NTP on the increased expression of MBP, NTP-mediated myelination was investigated using dorsal root ganglion (DRG)/SC co-culture system. DRG/SC co-cultures were maintained for 21 days, and the extent of myelination was quantified by measuring the number, length, and area of myelinated sections. NTP treatment showed no significant effects in the number of myelin segments on days 7, 14, and 21 after the induction of differentiation (Figure 4D,E). However, the mean length of MBP positive segments was significantly promoted in co-cultures containing NTP compared with control on days 14 and 21 (Figure 4D,F). In addition, the total area of MBP positive segments was significantly increased in NTP-containing co-cultures compared with control on days 14 and 21 (Figure 4D,G). Taken together, these results indicate that NTP promotes SCs differentiation and myelination in vitro.

### 2.5. NTP Promotes Remyelination and Functional Recovery in a Rat Lysophosphatidylcholine (LPC)-Induced Demyelination Model

**Figure 5:**
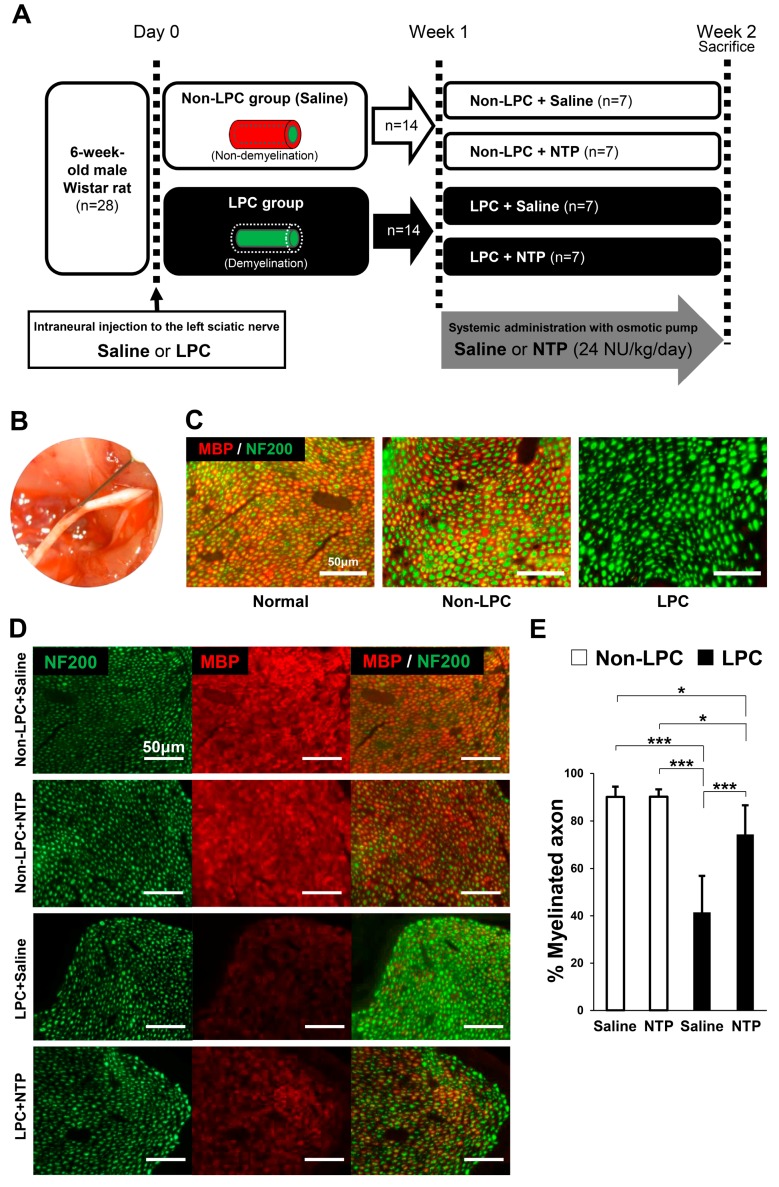
NTP accelerates remyelination following LPC-induced focal demyelination in vivo. (**A**) Experimental protocols are shown. (**B**) Photograph of saline (Non-LPC group; Non-demyelination) or LPC (LPC group; demyelination) injection to the left sciatic nerve. Intraneural injection was performed using a 30-gauge needle attached to a Hamilton syringe. One week after the injection, saline or NTP (24 NU/kg/day) were administered systemically with an osmotic pump for one week in both Non-LPC group and LPC group. Rats were divided into four groups: (1) Non-LPC + saline; (2) Non-LPC + NTP; (3) LPC + saline; (4) LPC + NTP. (**C**,**D**) Representative photomicrographs of transverse sections of sciatic nerves one week after saline (Non-LPC group) or LPC (LPC group) injection (**C**) and one week after the systemic administration of saline or NTP (two weeks after the injection to the sciatic nerve) (**D**). Sections were co-stained with MBP (red) and NF200 (green). Scale bar = 50 μm. (**E**) Quantification of myelinated axons (MBP positive axons) per total axons (NF200 positive axons) 1 week after the systemic administration of saline or NTP. Significance was determined by one-way ANOVA followed by the Tukey–Kramer HSD test. Graphs show mean ± SD (*n* = 7 for each group); * *p* < 0.05, *** *p* < 0.001.

In this study, a sciatic nerve focal demyelination model was made using LPC, which dissolves myelin sheaths, leading to pure demyelinated lesions that spontaneously remyelinate over time [25,26,27]. Focal demyelination reaches a peak about one week after the LPC injection into the sciatic nerve, and recovers almost completely in three weeks [25,28]. Considering the usefulness for clinical application, NTP was administered after completion of demyelination (one week after LPC injection). A previous report showed that it was possible to evaluate the effect of systemic drug administration for one week using this model [27]. Figure 5A shows the experimental protocols. One week after the injection of saline (Non-LPC group) or LPC (LPC group) into the sciatic nerve (Figure 5B), saline or NTP was administered systemically with an osmotic pump for one week. To identify the influence of LPC, demyelination was assessed by immunofluorescence of sciatic nerve cross-sections. One week after the intraneural injection, myelinated axons (MBP positive axons) were rarely observed in the LPC group, whereas they were clearly visible in the saline injected Non-LPC group (Figure 5C). Moreover, we confirmed that axonal damage due to needle insertion or LPC was absent in the sciatic nerves as abundant neurofilament 200 (NF200) positive axons in the LPC group were detected (Figure 5C). One week after systemic administration (two weeks after the intraneural injection), the ratio of MBP positive axons were estimated histologically. The ratio of MBP positive axons between the Non-LPC + saline group and the Non-LPC + NTP group were similar (Figure 5D,E) and the same as in normal sciatic nerve (Figure 5C). These findings suggest that NTP does not influence myelin in normal sciatic nerve. However, the ratio of myelinated to total axons was significantly decreased in the LPC + saline group compared with the Non-LPC + saline/NTP groups. The LPC + NTP group showed accelerated recovery of remyelination and the ratio of myelinated axons were approximately 1.8-fold in comparison with the LPC + saline group, even though this recovery was incomplete compared with the Non-LPC + saline/NTP groups (Figure 5D,E). These results demonstrate that NTP promotes remyelination in a rat LPC-induced demyelination model.

**Figure 6:**
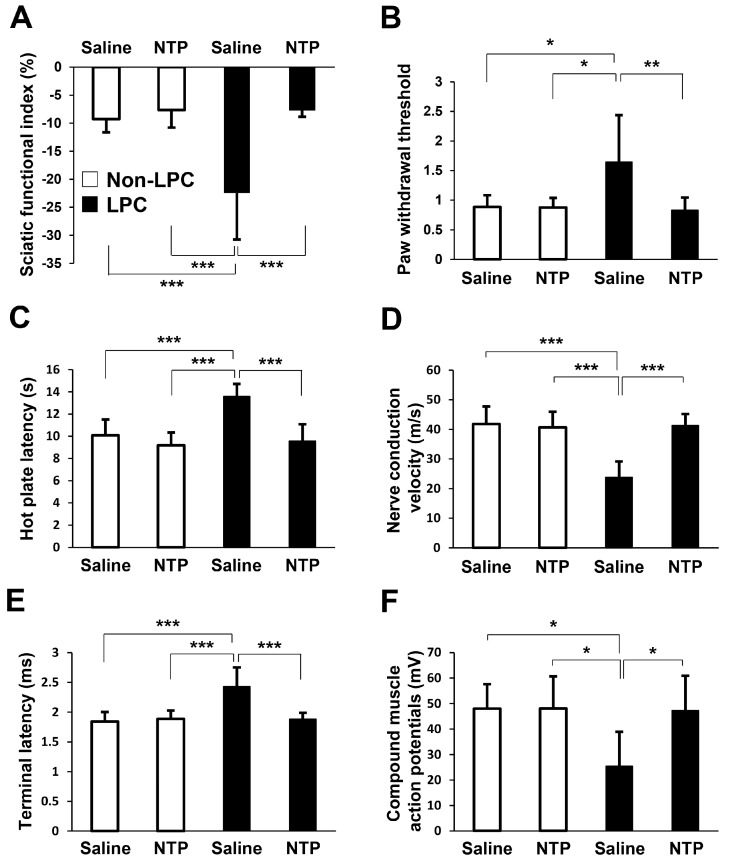
NTP prevents sciatic nerve dysfunction in a rat LPC-induced demyelination model. (**A**–**F**) To evaluate sciatic nerve functions, the sciatic functional index (SFI), von Frey filament test, and hot plate test, and electrophysiological analyses (NCV, TL, and CMAPs) were performed one week after the administration of saline or NTP (2 weeks after the injection to the sciatic nerve). Significance was determined by one-way ANOVA followed by the Tukey–Kramer HSD test. Graphs show mean ± SD (*n* = 7 for each group); * *p* < 0.05, ** *p* < 0.01, *** *p* < 0.001.

To evaluate motor functional recovery, we performed sciatic functional index (SFI) analysis on the rats. One week after systemic treatment, the SFI was significantly decreased in the LPC + saline group compared with the Non-LPC + saline/NTP groups. NTP treatment prevented the reduction of the SFI (Figure 6A). Sensory function was also measured by the von Frey filament and hot plate tests. Withdrawal thresholds to mechanical stimuli applied on the hind paw were determined using the von Frey filament test. Paw withdrawal thresholds were significantly higher in the LPC + saline group compared with the Non-LPC + saline/NTP groups, while NTP treatment prevented this LPC-induced deterioration of the mechanical thresholds (Figure 6B). The hot plate test was carried out to assess the response latency to a thermal stimulus applied to the hind paw. The response latency was significantly higher in the LPC + saline group in comparison with the Non-LPC + saline/NTP groups, while administration of NTP improved this response (Figure 6C). Furthermore, we performed electrophysiological evaluation to measure nerve conduction velocity (NCV), terminal latency (TL), and compound muscle action potentials (CMAPs). All of them were significantly deteriorated in the LPC + saline group compared with the Non-LPC + saline/NTP groups, while they significantly recovered to control levels with NTP treatment (Figure 6D–F). No statistically significant differences between the Non-LPC + saline and Non-LPC + NTP groups were observed (Figure 6A–F). These results demonstrate that NTP promotes neurological function recovery in a rat model of demyelination.

## 3. Discussion

This is the first report demonstrating the effect of NTP on nerve regeneration, to the best of our knowledge. Our results revealed that NTP promoted SCs differentiation in vitro and remyelination after LPC-induced focal demyelination in vivo.

The main constituents of the PNS are neurons and SCs, and after PNI, the isolated distal axons undergo Wallerian degeneration. SCs distal to the injury site dedifferentiate, proliferate, and line the endoneurial tubes to guide regenerating axons, finally re-differentiating to the myelination stage after guiding axons toward the target [29]. Previous studies on neurons demonstrated that NTP promotes neurite outgrowth in PC12 cells [30,31], and attenuates the inhibition of neurite outgrowth induced by paclitaxel or oxaliplatin in PC12 cells and DRG neurons [32,33]. Amelioration of demyelination in the chronic constriction injury model [15] and recovery in a paclitaxel-induced demyelination model [32] were also observed. These findings remind us of the possibility that NTP brings about a favorable effect in both neurons and SCs and provides a new therapeutic strategy, not only for the treatment of neuropathic pain, but also for nerve regeneration after PNI.

A lot of studies have provided evidence regarding the functions of AKT and MAPK in the regulation of SCs plasticity. The sustained activity of AKT signaling is crucial for initiation of SCs myelination, and the opposing functions of AKT and ERK1/2 pathways control SCs myelination [16]. Activities of MAPKs (ERK, p38, and JNK) are rapidly and highly enhanced in the SCs around the injury site after PNI, and play specific, overlapping or complementary roles in SCs plasticity [16,17,18,34,35]. Sustained ERK activation inhibits the transition from immature SCs to promyelinating SCs, acting as a dedifferentiation signal, and negatively modulates myelination [18]. In this study, NTP increased AKT activity and reduced ERK1/2 activity in SCs in both the growth and differentiation media (Figure 1 and Figure 2). Previous studies have demonstrated that c-JUN was upregulated through MAPK kinase (MKK)7/JNK [36] and/or MKK6/p38 [34]. There may be cross-talk between the JNK and p38 signaling pathways associated with c-JUN induction in SCs plasticity [18]. In our study, NTP inhibited the activation of p38, although JNK and c-JUN activities were unaffected (Figure 1C–E). These findings suggest that the inhibition of p38 activity would have no influence on c-JUN activity, due to the unchanged activity of the JNK/c-JUN axis after the addition of NTP. Therefore, we conclude that the axis of p38/c-JUN is not a main downstream target of NTP for SCs plasticity.

Concerning the differentiation of SCs, previous reports demonstrated that SCs express both P0 and MAG in the promyelinating stage and MBP in the myelinating stage [16,37,38]. Under growth conditions, NTP promoted the expression of P0 (Figure 4B), a promyelinating marker, with the activation of AKT (Figure 1A,F) and the inactivation of ERK (Figure 1B,G). These findings suggest that the administration of NTP might be an initiation step for SCs to transit from the proliferation stage to the differentiation stage, because sustained AKT activation and ERK inactivation is crucial for initiation of SCs differentiation. Although MAG is another promyelinating marker, its expression was not increased in SCs with NTP under the differentiation conditions (Figure 4C). We confirmed its expression in SCs cultivated for 72 h. The expression of MAG reached a peak at 36–48 h in differentiation medium lacking axons [38,39]. Accordingly, NTP might not increase the expression of MAG by SCs in the differentiation conditions. In the late stage of SCs differentiation, the expression of MBP becomes strong. NTP accelerated the expression of MBP in SCs under differentiation condition (Figure 4A), and in co-cultured DRG/SC in the late stage after differentiation but not in the early stage (Figure 4D–G). Moreover, NTP promoted the re-expression of MBP (remyelination) in the LPC-induced demyelination model (Figure 5C,D). Taken together, these findings indicate that NTP accelerates the differentiation of SCs in both the promyelinating and myelinating states. In our study, NTP upregulated Krox20 under both growth and differentiation conditions (Figure 1H and Figure 2C). However, NTP did not downregulated c-JUN activity (Figure 1E), although c-JUN is a negative regulator of Krox20 [40]. The reason for this discrepancy is unclear but may be related to the time point of evaluation. We evaluated c-JUN activity in SCs only at 60 min after the administration of NTP in growth medium. Further research would be required to reveal how NTP affect the time-dependent c-JUN activity. Several studies have reported that NRG1 is a key axonal signal controlling myelination in the PNS, via signaling through ErbB2/3 tyrosine kinase receptors [41]. Nrg1 binding to ErbB2/3 receptors results in the activation of intracellular signal transduction pathways including AKT and ERK signaling [21,22,23]. In this study, NTP did not affect ErbB2 and ErbB3 activities under both growth and differentiation conditions (Figure 1I,J and Figure 2D,E). NTP may affect receptors other than ErbB2/3, or may pass through cell membrane and influence intracellular signaling pathways directly. Further studies are needed in order to unravel how NTP affects intracellular signaling pathways.

In the case of PNI, SCs dedifferentiate, proliferate, and redifferentiate with axon contact. However, a previous study using cyclin D1 null mice demonstrated that the proliferation of SCs was not essential for functional recovery following PNI. Furthermore, newly generated SCs after the PNI were culled by apoptosis in the wild type littermates [42]. This finding supports our data that NTP promoted functional recovery in a rat LPC-induced demyelination model despite no influence of NTP on SCs proliferation.

LPC has been reported to be synthesized following nerve injury and converted to lysophosphatidic acid by autotaxin, subsequently leading to demyelination and neuropathic pain (allodynia and hyperalgesia) through an unknown process [43,44,45,46]. However, our data displayed the heightened paw withdrawal thresholds and thermal withdrawal latencies after LPC injection (Figure 6B,C). These results indicated that our models showed plantar hypalgesia, not hyperalgesia, similar to previous studies [27,47,48]. The cause for this discrepancy requires further investigation.

Our study has several limitations. First, a main active ingredient of NTP is unclear because NTP consists of so many components, including nucleic acids, amino acids, and sugars (unpublished observations). Second, the precise mechanisms of how NTP affects cell signaling in SCs remain to be elucidated. Third, NTP promoted remyelination in LPC-induced demyelination model. However, the evaluation of detailed myelin structure including myelin thickness using electron microscopy is not performed. Finally, it is unknown whether NTP can also be effectively applied to more severe cases, such as a rat sciatic nerve crush injury or a transection model. Future studies are needed to unravel these limitations.

In conclusion, NTP accelerates the differentiation of SCs without influencing their proliferation via AKT and ERK1/2 signaling in vitro and promotes remyelination and functional recovery in an LPC-induced demyelination model in rats. This indicates that NTP may be a novel treatment option for peripheral nerve injuries and demyelinating diseases.

## 4. Materials and Methods

### 4.1. Animals

Wistar rats (embryonic day 15, postnatal days 1–5, and 180–200 g adult; Oriental Yeast, Osaka, Japan) were utilized in this experiment. Animals were kept under a 12/12 h light/dark cycle (lights on 08:00–20:00 h) environment. All animals were allowed freely to access to food (MF, Oriental Yeast) and tap water. We performed all experiments in accordance with the National Institutes of Health Guide for the Care and Use of Laboratory Animals. They were approved by the Animal Care Committee of the Graduate School for Medicine, Osaka University (approval number: 24-026-010, approval date: 12 June 2012). We made maximum effort to minimize the number of animals utilized and to limit any suffering.

### 4.2. Drugs

NTP was provided from Nippon Zoki Pharmaceutical Co. (Osaka, Japan). The analgesic activity of NTP (expressed in NU) is standardized by a behavioral testing in rodents loaded with the “stress alteration of rhythm in environmental temperature”, a repeated cold stress by which hypersensitivity to a noxious stimulus is produced [49]. NTP does not contain detectable known proteins such as neurotrophins.

### 4.3. Primary SCs Culture

We prepared SCs from sciatic nerves of postnatal day 1–5 Wistar rats according to the procedure previously described [16,27]. In brief, SCs were seeded on poly-L-lysine-coated dishes (IWAKI, Shizuoka, Japan) with Dulbecco’s Modified Eagle’s Medium (DMEM; GIBCO/BRL Life Technologies, Grand Island, NY, USA) supplemented with 10% fetal bovine serum (FBS; Sigma-Aldrich, St. Louis, MO, USA). After 24 h of cultivation, selection using 10 μM cytosine arabinoside (Sigma-Aldrich) was performed to remove fibroblasts from SCs. Finally, SCs were cultured in growth medium (DMEM containing 3% FBS with 3 μM forskolin (Merck, Darmstadt, Germany) and 20 ng/mL of NRG (R&D Systems, Minneapolis, MN, USA)) in a humidified atmosphere of 5% CO_2_ at 37 °C. We obtained SCs cultures of >99% purity using these procedures. In all experiments, SCs were used between passages 3 and 8.

### 4.4. Western Blotting

Cultured SCs were homogenized with 100 μL Kaplan buffer (150 mM NaCl, 50 mM Tris-HCl (pH 7.4), 1% NP-40, 10% glycerol, and a protease inhibitor cocktail (Roche Diagnostics, Mannheim, Germany)). The lysate was subject to sodium dodecyl sulphate-polyacrylamide gel electrophoresis (SDS-PAGE) and Western blotting analysis with a standard procedure using primary antibodies against AKT (1:1000; CST 4691; Cell Signaling Technology, Beverly, MA, USA), phospho-AKT (1:1000; CST 4056; Cell Signaling Technology), ERK1/2 (1:1000; CST 4695; Cell Signaling Technology), phospho-ERK1/2 (1:1000; CST 9101; Cell Signaling Technology), p38 (1:1000; CST 9212; Cell Signaling Technology), phospho-p38 (1:1000; CST 9215; Cell Signaling Technology), JNK (1:1000; CST 9252; Cell Signaling Technology), phospho-JNK (1:1000; CST 4668; Cell Signaling Technology), c-JUN (1:1000; CST 9165; Cell Signaling Technology), phospho-c-JUN (1:1000; CST 3270; Cell Signaling Technology), Krox20 (1:1000; AV100880; Sigma-Aldrich), GAPDH (1:1000; CST 2118; Cell Signaling Technology), ErbB2 (1:1000; CST 4290; Cell Signaling Technology), phospho-ErbB2 (1:1000; CST 2247; Cell Signaling Technology), ErbB3 (1:1000; CST 12708; Cell Signaling Technology), phospho-ErbB3 (1:1000; CST 14525; Cell Signaling Technology), PCNA (1:1000; CST 2586; Cell Signaling Technology), MBP (1:1000; M3821; Sigma-Aldrich), P0 (1:1000; ab31851; Abcam, Cambridge, UK), and MAG (1:1000; MAB1567; Chemicon, Temecula, CA, USA) and secondary antibodies including a horseradish peroxidase-linked whole donkey anti-rabbit IgG (1:1000; NA934; GE Healthcare Life Sciences, Little Chalfont, UK). The integrated optical densities of protein bands were measured using ImageJ 1.45 s, which is a public-domain image analysis program developed at the U.S. National Institutes of Health (Rasband, W.S., ImageJ, U.S. National Institutes of Health, Bethesda, MD, USA, https://imagej.nih.gov/ij/, accessed on 21 January 2015).

### 4.5. BrdU Uptake Assay

Proliferation of SCs was evaluated using a BrdU uptake assay (Roche Diagnostics). Briefly, SCs were seeded into poly-l-lysine-coated 96 well plates (IWAKI) at a density of 1.0 × 10^4^ cells/well. Cells were left to adhere for 24 h prior to stimulation with NTP. Subsequently, the medium was changed to medium containing 10 μM BrdU, and cells were incubated for 2 h under standard cell culture conditions (37 °C and 5% CO_2_). The culture plates were fixated with FixDenat solution and incubated with anti-BrdU POD antibody solution for 90 min at room temperature. After washing the plate with phosphate-buffered saline (PBS), substrate solution (tetramethyl benzidine) was added for 30 min. Absorption was measured at 370 nm, with 492 nm as the reference wavelength, on an automatic microplate reader.

### 4.6. Cell Proliferation Assay

Cell proliferation ability was detected by cell counting as previously described with some modifications [27]. Briefly, SCs were seeded on poly-l-lysine-coated 60 mm dishes (IWAKI) at a density of 1.4 × 10^5^ cells in growth medium. Cells were left to adhere for 24 h prior to stimulation with NTP (0.5 NU/mL). On days 1, 3, 5, and 7 after treatment, cells were counted using the Countess Automated Cell Counter (Invitrogen, Carlsbad, CA, USA).

### 4.7. Assay for SCs Differentiation In Vitro

SCs were seeded on poly-L-lysine-coated 35 mm dishes (IWAKI) in growth medium. The following day, cells were treated with 1 mM db-cAMP (Sigma-Aldrich) to make differentiation medium [27,50,51], and cultivated for 72 h in differentiation medium with or without NTP (0.5 NU/mL). The effects of NTP on SCs differentiation were examined by western blotting using differentiation markers (MBP, P0, and MAG).

### 4.8. Dorsal Root Ganglion Neuron and Schwann Cell Co-Culture

The effects of NTP on SCs myelination in vitro were examined as previously described in detail [52]. Briefly, DRG neurons were harvested from the spinal cord of embryonic day 15 Wistar Rat. Cells were seeded on poly-l-lysine and laminin (Sigma-Aldrich)-coated 8-well chamber slides at a density of 40,000 cells/well in a defined culture medium containing neurobasal medium (Invitrogen) supplemented with 2% B27 nutrient supplement (Invitrogen), l-glutamine (0.2 mmol/L; Invitrogen), and nerve growth factor (50 ng/mL; Millipore, Billerica, MA, USA). After 7 days, the medium was changed to that supplemented with 50 μg/mL ascorbic acid to initiate basal lamina formation and myelination with or without NTP (0.5 NU/mL). Co-cultures were allowed to myelinate for 3 weeks, with half amount of fresh medium changed every day.

### 4.9. Immunocytochemistry

DRG/SC co-cultures in 8-well chamber slides were fixed in 4% paraformaldehyde for 30 min at room temperature. Subsequently, co-cultures were blocked with PBS containing 0.2% Triton X and 5% bovine serum albumin (Sigma-Aldrich) for 1 h, and incubated overnight with primary antibodies at 4 °C. Fluorescence-conjugated secondary antibodies were reacted for 2 h at room temperature. The primary antibodies were MBP (1:1000; NE1018; Calbiochem, SanDiego, CA, USA) and NF200 (1:1000; N4142; Sigma-Aldrich). The secondary antibodies were Alexa Fluor 488 goat anti-rabbit IgG (1:1000; A-11034; Molecular Probes, Eugene, OR, USA) and Alexa Fluor 568 goat anti-mouse IgG (1:1000; A-11004; Molecular Probes). The number, length, and area of MBP-positive segments were evaluated using NIS Elements BR software (Laboratory Imaging, Nikon, Tokyo, Japan).

### 4.10. Surgical Procedures

We used twenty-eight male Wistar rats weighing 180–220 g (Charles River Laboratories Japan, Inc., Yokohama, Japan) in this study. Upon all experiments, animals were deeply anesthetized using subcutaneous injection of mixture of midazolam (2 mg/kg), butorphanol (2.5 mg/kg), and medetomidine (0.15 mg/kg), and immobilized in the prone position. For sciatic nerve demyelination experiments, the left sciatic nerve was exposed and freed from surrounding tissues from the sciatic notch to its bifurcation into the tibial and peroneal nerves. In LPC group (demyelination group), 5 μL of 2% LPC (Sigma-Aldrich) in saline was injected into the sciatic nerve 5 mm distal to the sciatic notch via a 30-gauge Hamilton syringe, as previously described [25,26,27,43,53] (Figure 5B). In Non-LPC group (Non-demyelination group), an equal volume of saline was identically applied. The needles were left in the nerve for 2 min following injection to prevent leakage. After injection, muscles and skin were closed in layers. One week after the operation, an osmotic pump (Model 2ML1; Alzet, Cupertino, CA, USA) was placed subcutaneously in the back to deliver continuous saline or NTP (24 NU/kg/day) for 1 week in both Non-LPC group and LPC group, as previously described [27,54]. Rats were divided into four groups: (1) Non-LPC + saline; (2) Non-LPC + NTP; (3) LPC + saline; and (4) LPC + NTP (*n* = 7 for each group) (Figure 5A).

### 4.11. Histological Analysis

For histological evaluation of the lesions, animals were sacrificed 1 or 2 weeks after the LPC injection into the sciatic nerve. They were anesthetized and the sciatic nerve containing the area of LPC application was excised for the evaluation of the extent of demyelination and remyelination. Sciatic nerves were fixed in 4% paraformaldehyde for 24 h at room temperature and then stored in 20% sucrose in 0.01 M PBS. The tissues were embedded in the Frozen Section Media (Leica Biosystems, San Diego, CA, USA), frozen in liquid nitrogen, sectioned axially at 5 μm, and mounted on a glass slide. They were permeabilized with 100% methanol for 30 min at −20 °C. After blocking with PBS containing 0.2% Triton X and 5% bovine serum albumin, they were incubated with primary antibodies against MBP (1:1000; NE1018; Calbiochem) and NF200 (1:1000; N4142; Sigma-Aldrich) overnight at 4 °C inside a humidified chamber, followed by incubation with the appropriate secondary antibodies including Alexa Fluor 488 goat anti-rabbit IgG (1:1000; A-11034; Molecular Probes) and Alexa Fluor 568 goat anti-mouse IgG (1:1000; A-11004; Molecular Probes). The myelinated ratio was calculated as the number of both MBP- and NF200- positive axons (myelinated axons) to the number of NF-200 positive axons (total axons) using NIS Elements BR software (Laboratory Imaging, Nikon).

### 4.12. Sciatic Functional Index

To evaluate motor function, the SFI was calculated as previously described [55,56,57]. At 2 weeks after the intraneural injection, rats were made to walk across a narrow track. The hindpaws were dipped in black ink and footprints were recorded on white paper. SFI was calculated from the footprints according to the formula established. The following parameters were measured: print length (PL), which is the distance from the heel to the toe; toe spread (TS), which is the distance from the first to the fifth toes; and intermediary toe spread (ITS), which is the distance from the second to the fourth toes. PL, TS, and ITS were collected on both the normal (N) and the experimental (E) hind legs.

(1) SFI = −38.3 × (EPL − NPL)/NPL + 109.5 × (ETS − NTS)/NTS + 13.3 × (EITS − NITS)/NITS − 8.8

The SFI varies from 0 to −100: scores around 0 indicate normal nerve function and around −100 indicates complete loss of function.

### 4.13. Von Frey Filament Test

To evaluate sensory function, at 2 weeks after the operation, paw withdrawal thresholds were measured to assess mechanical sensitivity with calibrated von Frey filaments (TouchTest, North Coast Medical Inc., Gilroy, CA, USA) as previously described [58,59]. Rats were placed on elevated metal mesh and von Frey filaments were applied to the plantar surface of the hindpaw until they bent. Values were normalized to the unaffected side.

### 4.14. Hot Plate Test

The hot plate test was performed as previously described [60,61,62]. Rats were individually placed on a hot plate (Ugo Basile, Varese, Italy) at temperature of 52.5 °C. Latency to the first sign of paw licking or jump response to avoid thermal pain was taken as an index of pain threshold.

### 4.15. Electrophysiological Analysis

Electrophysiological analysis was performed at 2 weeks after the operation. A pair of stimulating electrodes was noninvasively placed on the proximal side to the LPC application. We placed a recording electrode in the tibialis anterior muscle to record the CMAPs and the TL. The NCV was calculated using two different points across the lesions. CMAPs, TL, and NCV were detected and measured using the PowerLab device and software (AD Instruments, Bella Vista, NSW, Australia).

### 4.16. Statistics

Data are expressed as mean ± standard deviation (SD). Statistical evaluation was performed by one-way analysis of variance (ANOVA) followed by a post hoc Student’s *t*-test, Dunnett’s test, or Tukey–Kramer HSD test using JMP software, version 11 (SAS Institute, Cary, NC, USA).

## Abbreviations

ANOVA	analysis of variance
BrdU	5-bromo-2-deoxyuridine
CMAPs	compound muscle action potentials
CNS	central nervous system
db-cAMP	dibutyryl cyclic AMP
DMEM	Dulbecco’s Modified Eagle’s Medium
DRG	dorsal root ganglion
ERK	extracellular signal-regulated kinase
FBS	fetal bovine serum
ITS	intermediary toe spread
JNK	c-Jun-*N*-terminal kinase
LPC	lysophosphatidylcholine
MAG	myelin-associated glycoprotein
MAPK	mitogen activated protein kinase
MBP	myelin basic protein
MKK	MAPK kinase
NCV	nerve conduction velocity
NF200	neurofilament 200
NRG	neuregulin
NTP	Neurotropin^®^
NU	Neurotropin Unit
P0	protein zero
PBS	phosphate-buffered saline
PCNA	proliferating cell nuclear antigen
PL	print length
PNI	peripheral nerve injury
PNS	peripheral nervous system
SCs	Schwann cells
SFI	sciatic functional index
TL	terminal latency
TS	toe spread
